# Rare Groin Hernias With Unusual Sac Contents: A Systematic Review of Anatomical Variants and Surgical Implications

**DOI:** 10.7759/cureus.116135

**Published:** 2026-09-12

**Authors:** Christos-Pavlos Filippou, Dimosthenis Chrysikos, Theodore Troupis, Dimitrios Filippou

**Affiliations:** 1 Department of Anatomy, School of Medicine, National and Kapodistrian University of Athens, Athens, GRC

**Keywords:** amyand hernia, de garengeot hernia, littré hernia, rare groin hernia, reduction en masse, sliding inguinal hernia, unusual sac contents

## Abstract

Groin hernias are commonly encountered in general surgical practice. Most of these hernias have a typical anatomical structure and contain the usual type of hernia sac content. Still, in some rare cases, a hernia may show abnormal anatomy or contain unexpected material within the sac, which can affect both how it presents clinically and the surgical approach needed for treatment. This systematic review seeks to give an overview of rare groin hernias with unusual sac contents, detail their anatomical variations, describe their clinical features and diagnostic difficulties, and discuss the implications that may come up during surgical management.

A systematic review with qualitative synthesis was performed according to the Preferred Reporting Items for Systematic Reviews and Meta-Analyses (PRISMA) guidelines. The search was carried out in PubMed/Medical Literature Analysis and Retrieval System Online (MEDLINE) and included studies on rare inguinal and femoral hernias in adult patients. A focused supplementary search was also performed for hernias containing Meckel’s diverticulum. Studies were considered eligible when they described unusual hernia sac contents or relevant anatomical variations. Pediatric studies, review articles without original data, retracted publications, studies unrelated to inguinal or femoral hernias, and studies without a clear description of sac contents were excluded. In total, 43 studies were included in the final qualitative synthesis.

Several uncommon inguinal and femoral hernias containing unexpected sac contents were found. The documented contents included the vermiform appendix, Meckel's diverticulum, segments of the colon, the urinary bladder, and bowel that became trapped after reduction. The main types described were Amyand hernia, De Garengeot hernia, Littré hernia, sliding inguinal hernia, and reduction en masse. Diagnosing these conditions before surgery was often challenging, since many cases looked like typical incarcerated or strangulated groin hernias. CT scans or ultrasonography were helpful in certain cases, but the diagnosis was usually established during the operation. The surgical approach depended on the sac's contents, the presence of inflammation or contamination, the viability of the bowel, and whether any organs were involved.

Inguinal and femoral hernias that contain unusual sac contents are uncommon, but the cases reviewed indicate that they can influence both diagnosis and surgical management. The hernia sac might hold the appendix, Meckel's diverticulum, colon, urinary bladder, or bowel that becomes trapped after what appears to be a reduction. Since the clinical symptoms frequently mimic those of a typical incarcerated or strangulated groin hernia, making a preoperative diagnosis is challenging, and many of these cases are only identified during surgery. The surgical approach should be tailored to the specific sac contents, the presence of inflammation or contamination, the viability of the bowel, whether the bladder or colon is involved, and the overall condition of the patient. As most of the available evidence comes from case reports and small case series, it is not possible to make strong treatment recommendations, and there is a need for more consistent reporting.

## Introduction and background

Groin hernias are frequently seen in general surgical practice. They often present in a typical anatomical form, with the contents of the hernia sac being largely predictable. However, there are occasional instances where anatomical variations or atypical contents are observed, significantly impacting both clinical presentation and surgical management [1-3].

A rare groin hernia is characterized by the presence of uncommon organs within the hernia sac, such as the appendix (vermiform), Meckel's diverticulum, segments of the colon, or even the bladder [2-5]. Notable examples include the Amyand hernia, De Garengeot hernia, Littré hernia, sliding inguinal hernia, and reduction en masse. For the purposes of this review, these entities are included within the spectrum of rare groin hernias because they feature unusual sac contents, involvement of visceral organs in the hernia sac wall, or persistent visceral entrapment after apparent reduction. Amyand and De Garengeot hernias involve the vermiform appendix in an inguinal and femoral hernia, respectively. Littré hernia involves Meckel's diverticulum. Sliding hernias are marked by visceral organs forming part of the sac wall, and reduction en masse refers to persistent bowel entrapment even after apparent hernia reduction. Although these hernias are rare, they present intriguing clinical cases due to their association with a higher risk of complications.

Preoperative identification of these rare hernia variants proves challenging, as patients often exhibit clinical signs similar to those of standard groin hernias [6-8]. Clinical presentation can involve a painful or irreducible groin swelling. In some cases, atypical or complicated presentations might show inflammatory changes, signs of bowel obstruction, or ongoing obstruction even after apparent hernia reduction. These findings are usually nonspecific and do not consistently identify the anatomical contents of the hernia before surgery. While advanced imaging techniques have enhanced the preoperative diagnosis of some cases, it remains difficult to accurately identify this type of hernia until it is discovered during surgery [4,6,7,9]. The presence of uncommon structures within the sac can lead to various complications (such as inflammation, incarceration, ischemia, and perforation), necessitating the formulation of a comprehensive surgical strategy by the surgeon [10-12].

Despite an increase in published case reports, the available literature remains scattered and consists largely of isolated information. This systematic review provides a qualitative anatomical and surgical summary of adult case reports and small case series that describe rare inguinal and femoral hernia types, focusing on anatomical variants, clinical presentation, diagnostic challenges, and surgical management as found in the existing literature. 

## Review

Materials and methods 

Study Design

This study is a systematic review with qualitative synthesis of published studies concerning rare inguinal and femoral hernias in adult patients. Special attention is given to atypical contents of hernia sacs and associated anatomical variations highlighted in the literature, including unexpected tissue findings and rare structural characteristics. The design and reporting of the study adhered to the Preferred Reporting Items for Systematic Reviews and Meta-Analyses (PRISMA) guidelines [13], which are recognized for promoting transparency and consistency in systematic reviews.

Information Sources

The literature search was performed in PubMed/Medical Literature Analysis and Retrieval System Online (MEDLINE). PubMed/MEDLINE was selected as the primary bibliographic source because of its broad coverage of peer-reviewed biomedical and surgical literature and its reproducible search functionality.

Search Strategy

The search was conducted using free-text terms combined with Boolean operators. No MeSH terms were used. The main search strategy was: (“Amyand hernia” OR “De Garengeot hernia” OR “Littre hernia” OR “Littré hernia” OR “Meckel diverticulum” OR “Meckel's diverticulum” OR “sliding inguinal hernia” OR “sliding hernia” OR “reduction en masse”) AND (inguinal OR femoral).

The main search was conducted on January 31, 2026, with the final literature-search cutoff set at February 26, 2026, following the supplementary search. The search was restricted to English-language publications during the 2021-2026 publication period, while adult eligibility was assessed during study screening. A focused supplementary search was performed for Littré hernia to maximize the retrieval of reports involving Meckel’s diverticulum. The supplementary search strategy was: (“Littre hernia” OR “Littré hernia”) AND (“Meckel diverticulum” OR “Meckel's diverticulum”) AND (inguinal OR femoral). This search identified nine candidate records; after comparison with the primary-search set and application of the eligibility criteria, four eligible reports were added to the final qualitative synthesis. The Littré search was the only entity-specific supplementary search performed; no separate supplementary searches were undertaken for Amyand hernia, De Garengeot hernia, sliding inguinal hernia, or reduction en masse beyond the primary search strategy.

Eligibility Criteria

Inclusion criteria: For a study to be included in the systematic review, it had to meet specific criteria, such as studies involving adult patients, describing rare inguinal or femoral hernias, reporting unusual hernia sac contents (e.g., vermiform appendix, Meckel’s diverticulum, or visceral organs in sliding hernias), providing full-text access or sufficient information through the abstract, and being published in English.

Exclusion criteria: Studies were excluded from the analysis if they involved solely pediatric populations, were unrelated to inguinal or femoral hernias, were review articles without original data, had been retracted, or lacked a clear description of hernia sac contents.

Study Selection

**Figure 1:**
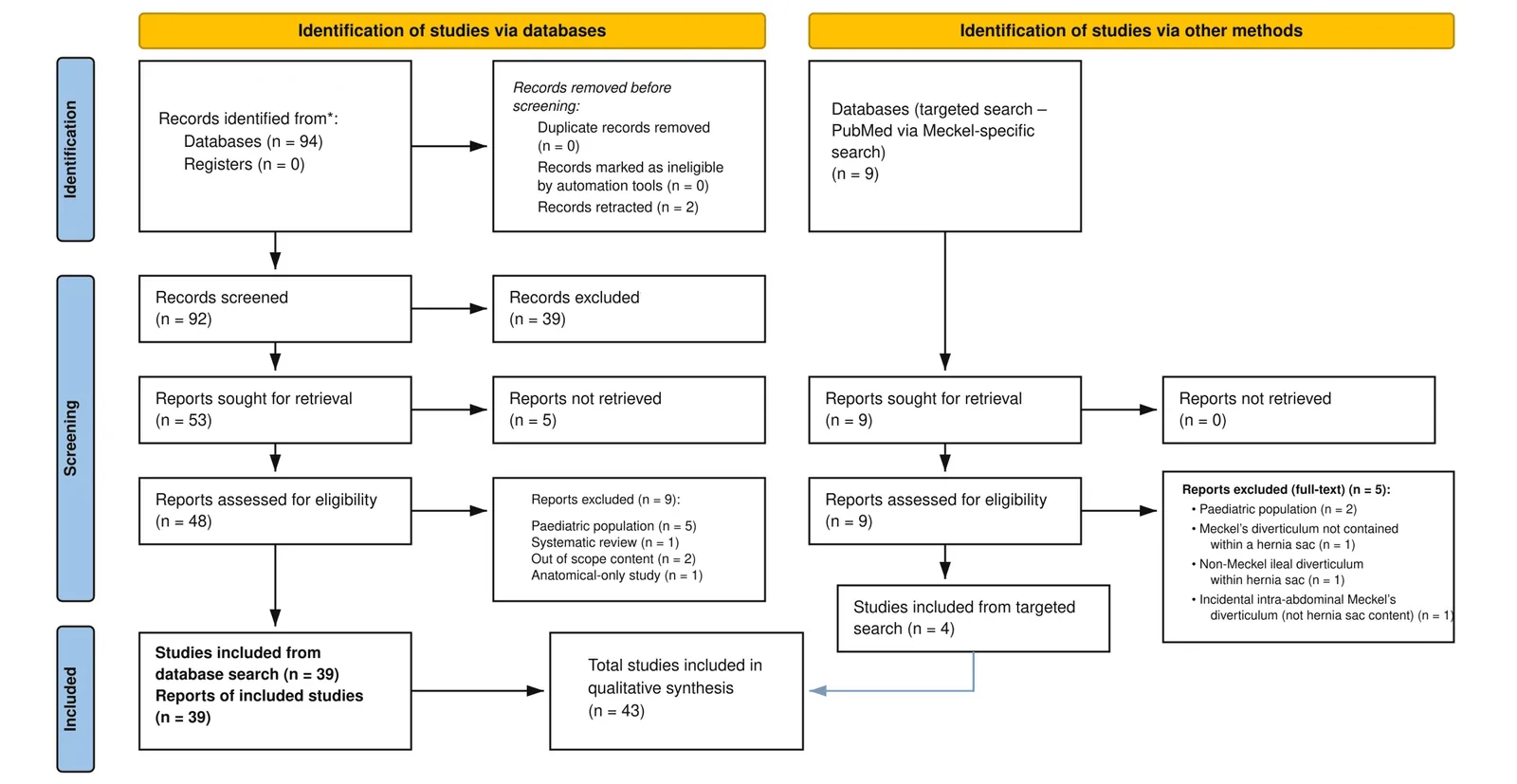
PRISMA 2020 flow diagram illustrating the identification, screening, eligibility assessment, and final inclusion of studies in the systematic review PRISMA: Preferred Reporting Items for Systematic Reviews and Meta-Analyses [13]

The initial literature search resulted in 94 records. Two retracted publications were identified and excluded before screening. The remaining 92 records underwent title and abstract screening independently by two reviewers, resulting in 53 reports selected for full-text retrieval. No duplicate records were identified. Any disagreements between the two reviewers were resolved by consensus. No formal inter-reviewer agreement statistic was calculated. Of these, five reports for which the full text could not be obtained were excluded before full-text eligibility assessment and were not included in the qualitative synthesis. A total of 48 studies were evaluated for full-text eligibility, with nine excluded based on the established criteria. Ultimately, 39 studies from the primary search and four additional studies from the supplementary search were included in the final qualitative synthesis of the review. The extracted data, results, and final manuscript were subsequently reviewed and evaluated by all authors. The identification, screening, eligibility assessment, and inclusion process is represented in the PRISMA flow diagram (Figure 1).

Data Extraction

From the studies meeting the inclusion criteria, data were gathered on patient demographics, clinical characteristics, hernia type, sac contents, surgical approaches, and, when available, postoperative outcomes. A predefined data collection form was used for data extraction to maintain consistency and comparability.

Quality Assessment

A formal risk-of-bias assessment was not performed because the included evidence consisted predominantly of heterogeneous case reports and case series.

Synthesis Methods

Because the included studies consisted mainly of case reports and small case series with substantial clinical and methodological heterogeneity, a quantitative meta-analysis was not feasible. Therefore, the findings were synthesized qualitatively. No pooled effect estimates, meta-regression analyses, or inferential statistical comparisons were performed. The findings were depicted, focusing on patterns observed across the included case reports and small case series, rather than providing comparative estimates of treatment effectiveness.

Results

Hernias Containing the Appendix

Amyand hernia: Refers to the presence of the vermiform appendix within the sac of an inguinal hernia [1]. In the studies included in the present review, the appendix was identified within the inguinal hernia sac, most commonly on the right side. In several cases, inflammatory changes of the appendix were reported, including acute appendicitis, while more advanced presentations included gangrenous changes or perforation of the appendix [2,14].

Preoperative diagnosis of an Amyand hernia was often challenging, as the clinical presentation frequently mimicked that of an incarcerated inguinal hernia. In many cases, the diagnosis was established intraoperatively during surgical repair of the hernia. In some cases, however, imaging techniques such as computed tomography or ultrasonography contributed to the preoperative identification of the appendix within the hernia sac [6,15].

Regarding surgical management, appendectomy was performed in cases where inflammatory changes or complications of the appendix were present. Hernia repair was performed either with mesh or with tissue-based techniques, depending primarily on the presence of inflammation or contamination of the operative field [1,6]. In some reports, appendiceal neoplasms such as neuroendocrine tumors or adenomas were identified following histopathological examination of the resected appendix [14,16].

De Garengeot hernia: Defined as the presence of the vermiform appendix within a femoral hernia sac [17,18]. In the included studies, most cases involved adult patients presenting with a painful, irreducible groin swelling. Right-sided localization was frequently reported, and the hernia sac often contained an inflamed appendix [7,19].

Preoperative diagnosis was frequently difficult because the clinical presentation closely resembled that of a strangulated femoral hernia [18]. Consequently, the diagnosis was often established intraoperatively during surgical exploration [11,20]. Nevertheless, in some cases preoperative imaging with computed tomography or ultrasonography allowed identification of the appendix within the femoral canal [7,21].

Surgical management most commonly involved appendectomy combined with femoral hernia repair. The choice of repair technique depended mainly on the presence of inflammation or contamination of the operative field. In reported cases with appendiceal perforation or severe inflammation, tissue repair without mesh was more frequently described [19,22], whereas mesh repair or laparoscopic techniques were reported in selected cases without significant inflammation [23,24]. Complications such as appendicitis, perforation, or gangrenous changes were reported, while rare cases included neoplastic lesions of the appendix within the hernia sac [12,25].

Hernias Containing Meckel’s Diverticulum (Littré Hernia)

Six studies describing adult patients with hernias containing Meckel’s diverticulum (Littré hernia) were included in the present review [5,26-30]. These cases primarily involved inguinal or femoral hernias in which Meckel’s diverticulum was identified intraoperatively as part of the hernia sac content. Clinically, patients commonly presented with symptoms of an incarcerated groin hernia, without specific findings indicating the presence of Meckel’s diverticulum prior to surgery [26-28].

Preoperative diagnosis was uncommon, as both clinical and radiological findings often resembled those of incarcerated small bowel. In most cases, the diagnosis was established during surgery when the diverticulum was identified within the hernia sac [26-28]. In some reports, computed tomography suggested the diagnosis by demonstrating a blind-ending tubular structure within the hernia sac [5,30].

When Meckel’s diverticulum was the only content of the hernia sac, intestinal obstruction was not always present despite incarceration. This finding has been attributed to the anatomical nature of the diverticulum as a blind pouch arising from the ileum, which may not directly compromise the intestinal lumen at the level of the hernia neck [5].

Surgical treatment included diverticulectomy or segmental small bowel resection depending on the presence of ischemia or necrosis, followed by repair of the hernia defect with or without mesh [27,30]. In some cases laparoscopic techniques were employed, allowing both identification of the diverticulum and simultaneous hernia repair [29].

Sliding Hernias

The included studies described cases of sliding inguinal hernias in which intra-abdominal organs formed part of the wall of the hernia sac. One report described a massive right-sided sliding inguinal hernia containing the cecum, terminal ileum, appendix, and ascending colon. The hernia was complicated by ischemia and perforation of the ascending colon with associated abscess formation. Surgical management included right hemicolectomy with ileostomy [10].

Another case described a direct left-sided sliding inguinal hernia involving the wall of the urinary bladder and part of the colon, which was treated with robotic-assisted surgical repair using mesh [3].

In addition, a case of sliding inguinal hernia with significant displacement of the urinary bladder was associated with postrenal acute kidney injury due to bilateral hydronephrosis and hydroureter. The diagnosis was established with pelvic computed tomography [4].

A giant sliding inguinal hernia with a long-standing clinical course was also reported and treated surgically with mesh repair [31]. Finally, a rare case of sliding inguinal hernia in a patient with Fournier gangrene was described, in which hernia repair was performed after infection control and surgical debridement [32].

Reduction en Masse

Reduction en masse is a rare complication of inguinal hernia in which the hernia appears to be reduced, but the bowel loop remains trapped within the hernia sac in the preperitoneal space [8].

In the cases included in this review, patients developed symptoms of intestinal obstruction following spontaneous or manual reduction of a previously known inguinal hernia. Clinical presentation typically included abdominal pain and signs of bowel obstruction despite the absence of a palpable hernia on physical examination [33,34].

Diagnosis was based on the clinical history of recent hernia reduction and confirmed with imaging, most commonly computed tomography, which demonstrated an incarcerated bowel loop within the preperitoneal hernia sac [8,34].

Emergency surgical exploration was performed in all cases. Both laparoscopic and open approaches were described, allowing reduction of the entrapped bowel and assessment of bowel viability. In some reports, laparoscopic management allowed successful reduction of the bowel loop and subsequent hernia repair [8,9].

Early diagnosis and surgical treatment were emphasized as critical in preventing serious complications, as persistent entrapment of the bowel may lead to ongoing intestinal obstruction or ischemia [33].

Anatomical Observations Based on Hernia Sac Contents

Analysis of the included cases revealed variability in the contents of the hernia sac and in the anatomical structures involved. In Littré hernias, Meckel’s diverticulum was identified within the hernia sac of inguinal or femoral hernias and described intraoperatively as a blind tubular structure arising from the ileum [26-28]. In some reports, the diverticulum was located within the femoral canal without an associated small bowel loop and without signs of intestinal obstruction [5].

In sliding hernias, part of an intra-abdominal organ such as the colon or urinary bladder formed a component of the hernia sac wall [3,4]. Cases included large inguinal hernias containing segments of the right colon [10] and direct sliding hernias involving the bladder wall [3]. Displacement of the urinary bladder into the inguinal canal was also associated with hydronephrosis and hydroureter due to extrinsic ureteral compression [4].

In cases of reduction en masse, the hernia sac and the incarcerated bowel segment were displaced into the preperitoneal space after apparent reduction of the hernia [8,34]. Persistent bowel entrapment within the sac resulted in ongoing intestinal obstruction despite the absence of an external groin swelling [33]. The location of the entrapped bowel in the preperitoneal space was confirmed either radiologically or during laparoscopic exploration [9]. Additional reports described complicated appendix-containing groin hernias, including Amyand and De Garengeot hernias [35-39].

The main anatomical variants, diagnostic pitfalls, and management-changing factors identified in the included studies are summarized in Table 1.

**Table 1:** Anatomy-to-management matrix of rare groin hernias with unusual sac contents

Hernia entity	Key anatomical finding	Diagnostic trap	Intraoperative risk	Management-changing factor	Reported operative pattern	References
Amyand hernia	Vermiform appendix within an inguinal hernia sac	Mimics incarcerated inguinal hernia	Unexpected appendiceal inflammation, gangrene, perforation, or neoplastic finding	Appendix status and contamination	Appendectomy was reported for inflamed or complicated appendices; mesh use varied with contamination.	[1,2,6,14-16,38,39]
De Garengeot hernia	Vermiform appendix within a femoral hernia sac	Mimics strangulated femoral hernia	Appendiceal incarceration, appendicitis, gangrene, perforation, or rare neoplasm	Degree of inflammation, perforation, and contamination	Appendectomy with femoral repair was commonly reported; tissue repair was more frequently described in contaminated fields, while mesh or biologic mesh was reported in selected cases.	[7,11,12,17-25,35-37,40-43]
Littré hernia	Meckel’s diverticulum within an inguinal or femoral hernia sac	May resemble incarcerated bowel; obstruction may be absent	Ischemia or necrosis of Meckel’s diverticulum or adjacent bowel	Viability of diverticulum and bowel	Diverticulectomy or segmental bowel resection; hernia repair individualized	[5,26-30]
Sliding inguinal hernia	Colon or urinary bladder forms part of the hernia sac wall	Organ involvement may be missed preoperatively	Bladder or bowel injury during dissection	Recognition of sliding component	Careful dissection; open, laparoscopic, robotic, or hybrid repair depending on anatomy and contamination	[3,4,10,31,32]
Reduction en masse	Bowel remains trapped within the preperitoneal hernia sac after apparent reduction	Groin swelling disappears while obstruction persists	Delayed diagnosis with persistent obstruction or ischemia	History of recent reduction and CT findings	Urgent exploration, reduction of trapped bowel, viability assessment, and hernia repair	[8,9,33,34,44]

The 43 included studies comprised eight reports of Amyand hernia, 19 of De Garengeot hernia, six of Littré hernia, five of sliding hernia, and five of reduction en masse. The detailed characteristics of the 43 included studies are summarized in Table 2.

**Table 2:** Characteristics of the included studies Data are presented as reported in the source articles; no unreported values were inferred. ProGrip™ mesh (Medtronic, Minneapolis, MN, USA); Surgisis® mesh (Cook Biotech Inc., West Lafayette, IN, USA); 3DMax™ light mesh (Becton, Dickinson and Company, Franklin Lakes, NJ, USA); Phasix™ mesh (Becton, Dickinson and Company, Franklin Lakes, NJ, USA) POD: postoperative day; SBO: small bowel obstruction; TAPP: transabdominal preperitoneal; SIS: small intestinal submucosa; SSI: surgical-site infection; DGH: De Garengeot hernia; NR: not reported; N/A: not applicable

Reference	Author	Year	Country	Age/Sex	Hernia entity	Hernia type	Sac content	Preoperative diagnosis	Imaging modality	Operative approach	Additional procedure	Mesh use	Contamination status	Complications	Follow-up
[1]	Ghattas et al.	2024	Lebanon	64 M; 60 M	Amyand hernia	Right indirect inguinal/inguinoscrotal Amyand hernia	Appendix: inflammatory/congestive changes in both cases	No – Amyand diagnosis established intraoperatively in both cases	CT (case 1); US (case 2)	Open inguinal repair	Appendectomy in both; contralateral hernia repair also performed in case 2	Yes – Lichtenstein mesh	No gross contamination or abdominal sepsis reported	No reported postoperative complication	2-week follow-up unremarkable
[2]	Caruso et al.	2021	Italy	80 M	Amyand hernia	Right incarcerated indirect inguinoscrotal Amyand hernia	Perforated gangrenous appendix with periappendiceal abscess	No – preoperative diagnosis was incarcerated inguinoscrotal hernia	NR	Emergency open inguinal surgery under local anesthesia	Appendectomy; abscess drainage/lavage; drains	No – Bassini tissue repair	Yes – perforation and periappendiceal abscess	No reported postoperative complications	Discharged after 48 h; no wound complication/recurrence at 10 d, 1 mo, and 6 mo
[3]	Varghese et al.	2024	USA	85 M	Sliding inguinal hernia	Left direct sliding inguinal hernia	Bladder wall; colon also reported in the article title	Yes – direct sliding inguinal hernia recognized preoperatively	CT	Robotic repair → open reduction → robotic completion	Reduction/dissection of bladder from sac	Yes – 10×15 cm ProGrip polyester mesh	No contamination reported	Yes – postoperative ileus and scrotal swelling	Discharged POD4; long-term follow-up NR
[4]	Vincent et al.	2022	Canada	92 M	Sliding inguinal hernia	Left sliding inguinal bladder hernia	Most of the urinary bladder	Yes – imaging diagnosis	Pelvic US + CT	Nonoperative management because of high surgical risk	Bilateral nephrostomy tubes + indwelling Foley catheter	N/A	No operative contamination	Yes – presenting postrenal AKI with bilateral hydronephrosis/hydroureter	6 months: no further hernia/urinary complications
[5]	Kehagias et al.	2021	Greece	51 F	Littré hernia	Right femoral Littré hernia	Incarcerated Meckel diverticulum with reversible ischemia	Yes – Littré hernia suspected preoperatively	CT + abdominal radiography	Emergency open low-groin approach	Stapled Meckel diverticulectomy	Yes – mesh plug	No perforation or gross contamination reported	No reported postoperative complication	Discharged POD4; long-term follow-up NR
[6]	Radboy et al.	2023	Iran	48 M	Amyand hernia	Right inguinal Amyand hernia	Inflamed appendix	Yes – Amyand hernia diagnosed preoperatively	US	Laparoscopic	Appendectomy	No at index operation – hernia repair deferred	Acute appendicitis; no purulent peritoneal contamination described	No reported postoperative complication	Discharged POD2; later hernioplasty planned
[7]	Hayrapetyan et al.	2025	USA	63 F	De Garengeot hernia	Right femoral De Garengeot hernia	Inflamed distal appendix	Partial – appendix identified preoperatively but initially localized to the canal of Nuck; DGH confirmed intraoperatively	US + CT	Urgent robot-assisted surgery	Appendectomy + femoral hernia repair	Yes – preperitoneal mesh	No perforation or abscess reported	No reported postoperative complication	NR
[8]	Cross et al.	2024	USA	73 M	Reduction en masse	Right inguinal reduction en masse	~15 cm incarcerated small-bowel segment	Yes – reduction en masse suspected/diagnosed preoperatively	CT	Urgent laparoscopy; definitive open inguinal repair subsequently	Reduction of bowel; no bowel resection	Yes – Lichtenstein mesh repair	No – bowel viable, no contamination reported	No reported postoperative complication	Discharged without complications; long-term follow-up NR
[9]	Oshidari et al.	2022	Iran	48 M	Reduction en masse	Left indirect inguinal reduction en masse	Incarcerated jejunal loop	Yes – reduction en masse suspected/diagnosed preoperatively	US + CT	Exploratory laparoscopy → TAPP	Release of incarcerated bowel; no resection	Yes – polypropylene mesh	No – bowel viable, no contamination reported	No reported postoperative complication	Discharged POD2; long-term follow-up NR
[10]	Ahmed et al.	2025	UK	75 M	Sliding inguinal hernia	Massive right sliding inguinoscrotal hernia	Terminal ileum, cecum, appendix, ascending colon, and omentum	Yes – CT identified perforated ascending colon with abscesses within the hernia	CT abdomen/pelvis	Exploratory laparotomy	Right hemicolectomy + ileostomy + abscess drainage + hydrocelectomy	NR	Yes – colonic perforation and abscess	Yes – grade 1 surgical-site infection	ICU 2 d; discharged after ~1 week; long-term follow-up NR
[11]	Bhattarai et al.	2024	Nepal	85 M	De Garengeot hernia	Right femoral De Garengeot hernia	Perforated necrotic appendix	Yes – De Garengeot hernia identified preoperatively	Contrast-enhanced CT	Emergency open femoral repair	Appendectomy	No – nonabsorbable suture closure	Yes – perforation with ~20 mL purulent fluid	No reported postoperative complication	Discharged POD3; 2 weeks: healthy wound, no recurrence
[12]	Quach et al.	2025	USA	51 M	De Garengeot hernia	Right femoral De Garengeot hernia	Strangulated gangrenous appendix	No – CT suggested Amyand hernia; De Garengeot diagnosed intraoperatively	CT	Laparoscopic appendectomy + femoral hernia repair	Appendectomy	No prosthetic mesh reported – primary repair	Gangrenous appendicitis; no explicit perforation reported	No reported postoperative complication	Discharged POD2; long-term follow-up NR
[14]	Zouki & Dharmawardhane	2024	Australia	15 M	Amyand hernia	Right indirect inguinal Amyand hernia	Inflamed appendix; appendiceal neuroendocrine tumor (pT4) with serosal involvement	No – clinical diagnosis was acute appendicitis; Amyand hernia identified intraoperatively	None preoperatively	Laparoscopic appendectomy	Appendectomy; later cecectomy for involved proximal margin	No – right inguinal hernia repair was not performed	Macroscopic appendicitis; no perforation or intra-abdominal free fluid	C. difficile infection treated after readmission; no intra-abdominal collection	Discharged next morning; later cecectomy showed no residual malignancy; colonoscopy at 1 month unremarkable; 6-month review unremarkable
[15]	Sadeghi et al.	2024	USA	46 M	Amyand hernia	Right inguinal Amyand hernia	Vermiform appendix	Yes – CT confirmed Amyand hernia	CT	NR	NR	NR	Appendix described without inflammation; operative contamination NR	NR	NR
[16]	McNamee et al.	2023	USA	85 M	Amyand hernia	Incarcerated inguinal Amyand hernia	Appendix with acute appendicitis; serrated adenoma on pathology	NR	NR	Laparoscopic appendectomy	Interval inguinal hernia repair; exact repair technique NR	NR	Acute appendicitis; exact contamination status NR	NR	NR
[17]	Salawu et al.	2025	UK	71 F	De Garengeot hernia	Incarcerated femoral De Garengeot hernia	Inflamed/gangrenous appendix	NR / not clearly established preoperatively	NR	Open femoral approach + laparoscopic appendectomy	Appendectomy	No – McVay suture repair	Yes – inflamed/gangrenous appendix with contamination concern	No reported postoperative complication	Discharged after ~24 h; longer follow-up NR
[18]	Aljohani	2026	Saudi Arabia	31 M	De Garengeot hernia	Right femoral De Garengeot hernia	Markedly inflamed appendix	No – CT diagnosed acute appendicitis; DGH recognized intraoperatively	Contrast-enhanced CT	Diagnostic laparoscopy	Laparoscopic appendectomy; hernia repair deferred	No at index operation – repair deferred	Acute inflammation; no perforation described	No reported postoperative complication	Discharged POD1; elective laparoscopic femoral repair planned
[19]	West et al.	2025	UK	F, 70s	De Garengeot hernia	Right femoral De Garengeot hernia	Appendix with inflammation	Yes – CT confirmed De Garengeot hernia with appendiceal inflammation	CT	Laparoscopic appendectomy + open low-approach femoral herniorrhaphy	Appendectomy	No – primary suture repair	Yes – contamination/inflammation concern; explicitly guided avoidance of mesh	NR	NR
[20]	Tiwana & Kabir	2024	UK	85 F	De Garengeot hernia	Right femoral De Garengeot hernia	Normal appendix + fatty tissue	No – incidental intraoperative De Garengeot finding	US + CT	Laparoscopic femoral hernia repair	Appendix reduced; no appendectomy	Yes – absorbable mesh plug	No – appendix not inflamed; no contamination	No reported postoperative complication	Same-day discharge; long-term follow-up NR
[21]	Alkhalifa et al.	2024	Saudi Arabia	40 F	De Garengeot hernia	Right femoral De Garengeot hernia	Inflamed appendix	Yes – CT diagnosed femoral hernia containing an inflamed appendix	Contrast-enhanced CT	Open infra-inguinal (Lockwood) approach	Appendectomy	No – herniorrhaphy without mesh	Inflamed appendix and dusky hernia sac; no abscess/perforation reported	No reported postoperative complication	Good postoperative recovery; exact duration NR
[22]	Huynh & Strauss	2025	Australia	73 F	De Garengeot hernia	Right femoral De Garengeot hernia	Inflamed appendix with necrotic tip	Yes – De Garengeot hernia with acute appendicitis diagnosed preoperatively	CT	Laparoscopic appendectomy + open femoral repair	Appendectomy	No – primary suture repair	Yes – contaminated/inflamed field	No reported postoperative complication	Discharged POD1; satisfactory outpatient review
[23]	Mushtaq et al.	2024	USA	58 F	De Garengeot hernia	Right femoral De Garengeot hernia	Inflamed, nonperforated appendix	Yes – CT identified appendicitis within femoral hernia	CT	Combined open inguinal/femoral + laparoscopic approach	Laparoscopic appendectomy	Yes – biologic mesh	Inflamed but nonperforated appendix; no gross contamination reported	No reported postoperative complication	Discharged POD1; long-term follow-up NR
[24]	Song et al.	2025	China	69 F	De Garengeot hernia	Right femoral De Garengeot hernia	Incarcerated inflamed/ischemic appendix	Yes – De Garengeot hernia with appendicitis diagnosed preoperatively	CT + US	Fully laparoscopic TAPP	Laparoscopic appendectomy	Yes – 10×15 cm porcine SIS biologic mesh (Surgisis)	Inflamed/ischemic appendix; no perforation or peritonitis	No reported postoperative complications	Discharged POD6; 18 months: no recurrence
[25]	Murad et al.	2024	UK	62 F	De Garengeot hernia	Right femoral De Garengeot hernia	Inflamed appendix; low-grade appendiceal mucinous neoplasm on histology	No – CT interpreted lesion as a right inguinal hernia containing fluid	CT	Open groin exploration	Appendectomy; later right hemicolectomy	No – suture repair of femoral canal	Inflamed appendix; perforation/gross contamination NR	No adverse postoperative outcome reported	Subsequent hemicolectomy without adverse histologic features; surveillance program
[26]	Essobiyou et al.	2022	Togo	67 M	Littré hernia	Right inguinoscrotal Littré hernia	Strangulated Meckel diverticulum with necrotic ileal involvement	No – preoperative diagnosis was strangulated inguinoscrotal hernia	NR	Emergency open surgery	Meckel/bowel resection + ileo-ileal anastomosis	No – Shouldice tissue repair	Necrotic bowel; perforation/gross contamination not reported	No reported postoperative complication	Discharged POD6; good at 3 months; returned to work at 4 months
[27]	Johnson et al.	2021	Canada	39 M	Littré hernia	Right indirect inguinal Littré hernia	Long small-bowel segment + large Meckel diverticulum; ~20 cm necrotic bowel	No – preoperative diagnosis was incarcerated/strangulated inguinal hernia	None – urgent clinical diagnosis	Open inguinal approach → lower midline laparotomy	Bowel/Meckel resection + stapled side-to-side anastomosis	Yes – Bassini repair reinforced with absorbable Vicryl mesh	Necrotic bowel; no perforation reported	No reported postoperative complication	Discharged POD2; well at 6 weeks
[28]	Khamas & Salih	2025	Iraq	66 M	Littré hernia	Right direct inguinal Littré hernia	Strangulated Meckel diverticulum	No – preoperative diagnosis was strangulated direct inguinal hernia	US	Emergency open inguinal surgery	Meckel resection + end-to-end anastomosis	No prosthetic mesh – nylon-darn herniorrhaphy	Strangulation; perforation/gross contamination not reported	No reported postoperative complication	Discharged POD4; asymptomatic at 1 year
[29]	Nakayama et al.	2025	Japan	54 M	Littré hernia	Right inguinal Littré hernia	Meckel diverticulum	No – CT diagnosed incarcerated inguinal hernia; Meckel identified intraoperatively	CT	Emergency laparoscopic TAPP	Meckel diverticulectomy/diverticulotomy via small abdominal incision	Yes – 3DMax Light Mesh	No fecal contamination described; bowel resection separated from mesh field	No reported postoperative complication	Discharged POD8; 6 months: no recurrence
[30]	Yu & Ip	2025	Australia	F, 90s	Littré hernia	Femoral Littré hernia	Strangulated Meckel diverticulum + ischemic small bowel	Partial – CT showed strangulation; definite preoperative Littré diagnosis not documented	CT	Emergency operation; exact hernia repair approach NR	Small-bowel/Meckel resection + side-to-side anastomosis	NR	Ischemia/strangulation; perforation NR	No reported postoperative complication; recovered well	Exact follow-up duration NR
[31]	Davis et al.	2021	USA	39 M	Sliding inguinal hernia	Giant left sliding inguinal hernia	Ascending colon + left ureter + large fluid component	Yes – giant sliding inguinal hernia/anatomical involvement recognized preoperatively	US + CT	Open inguinal approach + preperitoneal assistance	Orchiectomy + scrotoplasty + US-guided aspiration of 3.9 L	Yes – 6×8 cm Phasix mesh, Lichtenstein fashion	No infection/contamination reported	No reported postoperative complication	Uncomplicated recovery; returned to work at 6 weeks
[32]	Kundan et al.	2021	India	50 M	Sliding inguinal hernia	Left irreducible sliding inguinal hernia	Cecum	No – sliding cecal component diagnosed intraoperatively	NR	Open anatomical hernia repair after infection control	Serial debridement for Fournier gangrene before hernia repair	NR	Fournier gangrene initially; hernia repair performed after infection control	No reported complication after hernia repair	2 weeks and 3 months: no complaints
[33]	Al-Jumaili et al.	2025	Denmark	60 F	Reduction en masse	Right inguinal reduction en masse	Incarcerated small-bowel segment	No – CT showed persistent SBO; reduction en masse confirmed at laparoscopy	CT	Diagnostic/therapeutic laparoscopy	Bowel released; peritoneal defect closed; no bowel resection	No at index surgery – definitive hernia repair deferred	No necrosis or perforation reported	No reported postoperative complication	Discharged POD2; elective definitive hernia repair planned
[34]	Najjari et al.	2021	Iran	50 M	Reduction en masse	Right inguinal reduction en masse	~5 cm incarcerated small-bowel loop	Yes – diagnosis based on clinical history + CT	CT	Midline laparotomy → open inguinal repair	Bowel released; no resection	Yes – Lichtenstein mesh	Slight bowel discoloration resolved; no contamination reported	No reported postoperative complication	Observed 5 days; discharged without complications; long-term follow-up NR
[35]	Taylor & Burton	2025	New Zealand	60 F	De Garengeot hernia	Right femoral De Garengeot hernia	Grossly inflamed/ischemic appendix	No – US/CT reported canal-of-Nuck pathology/Amyand hernia; DGH confirmed intraoperatively	US ×2 + CT	Open high approach / open femoral repair	Open appendectomy	No – primary suture closure with 1-0 Ethibond	Inflamed/ischemic appendix; no perforation or gross contamination reported	No postoperative complication at 3 weeks	Discharged POD1; complication-free at 3 weeks
[36]	Brain & Barsoom	2025	USA	55 F	De Garengeot hernia	Right femoral De Garengeot hernia	Perforated/necrotic appendix with abscess/purulence	No – CT/US suggested incarcerated femoral hernia with strangulated fat/cellulitis	CT + US	Robotic exploration/appendectomy + incision over femoral canal	Appendectomy; drainage; Penrose drain; definitive hernia repair deferred	No – mesh repair deferred	Yes – purulent/significant contamination	No reported postoperative complication	Discharged POD2; drain removed POD10; wound satisfactory at ~3 weeks; elective repair planned
[37]	Alghueryafy & Albakheet	2024	Saudi Arabia	87 F	De Garengeot hernia	Right femoral De Garengeot hernia	Perforated/gangrenous appendix + ~15 cm perforated gangrenous ileum	No – CT suggested incarcerated femoral hernia with strangulated small bowel; appendix not seen	CT	Emergency open femoral repair	Appendectomy + small-bowel resection + side-to-side stapled anastomosis	No – primary McVay tissue repair	Yes – perforated appendix and perforated gangrenous small bowel	Yes – postoperative ileus and wound seroma	Discharged POD7; 2-week wound well healed, no infection/other complication
[38]	Ali et al. (adult subgroup only)	2021	Ghana	n=10 adults; 26–76 y; 8 M/2 F	Amyand hernia	Complicated inguinal/inguinoscrotal Amyand hernias	Adult cases: inflamed/perforated appendix; some with gangrenous bowel and additional bowel/bladder contents	No – all diagnoses made intraoperatively	None – no diagnostic imaging	Open groin surgery; additional midline laparotomy in selected complicated adult cases	Appendectomy; bowel resection/anastomosis when required	No prosthetic mesh – nylon-darn tissue repair	Adult subgroup includes inflamed, perforated, and gangrenous/contaminated presentations	Yes – SSI, scrotal hematoma/collection, ileus, and groin pain occurred in the adult subgroup; no mortality	Overall series: no recurrence at ~1-year average follow-up; adult-specific follow-up not separately reported
[39]	Gbegli et al.	2024	UK	84 F	Amyand hernia	Right inguinal Amyand hernia	Perforated appendix + groin abscess; appendiceal adenocarcinoma on histology	Yes – after initial cellulitis/abscess impression, CT identified perforated Amyand hernia	US + CT	Open surgery	Appendectomy + right inguinal hernia repair	NR	Yes – appendiceal perforation and abscess	NR	Staging CT showed no metastatic disease; exact follow-up duration NR
[40]	Yu et al.	2024	Taiwan	56 F	De Garengeot hernia	Right femoral De Garengeot hernia	Incarcerated appendix with distal gangrenous change	Yes – preoperative imaging diagnosis	CT + US	Laparoscopic/transabdominal approach	Appendectomy + primary suture closure of femoral defect	No – no mesh	Acute suppurative appendicitis/serositis with fluid collection; no rupture described	No reported postoperative complication	Discharged POD4; 2 weeks: healed wound, no groin mass
[41]	MacConnell & Stein	2025	Australia	84 F	De Garengeot hernia	Right femoral De Garengeot hernia	Inflamed appendix	Yes – CT identified inflamed appendix within femoral hernia	CT	Emergency open high McEvedy approach	Appendectomy	No – primary suture closure	Acute appendicitis; no perforation or drainable collection	No reported postoperative complication	Discharged POD1; 6 weeks: healed wound, no recurrence
[42]	Iovino et al.	2024	Italy	72 M	De Garengeot hernia	Right femoral De Garengeot hernia	Inflamed appendix + omental fat	Yes – De Garengeot hernia identified preoperatively	US + CT	Open right crural approach	Appendectomy	Yes – polypropylene mesh plug	Inflammation present; gross contamination NR	No reported postoperative complication	Discharged after a few days; long-term follow-up NR
[43]	Mridha et al.	2025	UK	79 F	De Garengeot hernia	Right femoral De Garengeot hernia	Cecum + macroscopically normal appendix	No – preoperative diagnosis was femoral hernia; DGH incidental intraoperatively	US	Elective open femoral repair	Appendix/cecum reduced; no appendectomy	No – suture closure	No inflammation or contamination	No reported postoperative complication	Same-day discharge; long-term follow-up NR
[44]	Najmaddini et al.	2024	Iran	74 M	Reduction en masse	Right inguinal reduction en masse	Incarcerated bowel within reduced hernia sac	Yes – reduction en masse diagnosed preoperatively	CT	Open surgery	Release/reduction of sac and bowel; abdominal/peritoneal defects repaired	No – no mesh	No ischemia reported; contamination not described	No reported postoperative complications	12 months: no recurrence or complications

Discussion

Groin hernias with unexpected contents in the sac are uncommon, yet their significance goes beyond just being rare. In clinical practice, the specific anatomy can affect both how the hernia is diagnosed and how the operation is planned. When performing a standard inguinal hernia repair, surgeons generally anticipate finding bowel or omentum within the sac. However, the cases discussed in this review demonstrate that other structures can also be present, such as the appendix, Meckel's diverticulum, colon, urinary bladder, or bowel that stays trapped even after what appears to be a successful reduction. This becomes especially important in emergency situations, where the ultimate surgical approach is often shaped by what is discovered during the operation, including factors like inflammation, contamination, the health of the bowel, or the risk of harming an involved organ.

The literature available indicates that anatomical differences in rare groin hernias may shape both the diagnostic process and decisions made during surgery. Still, this observation should be treated carefully, since most of the studies considered are either case reports or involve only a few cases. As a result, the conclusions from this review should not be taken as final guidelines for treatment. Instead, their main contribution is in gathering information about anatomical variations, diagnostic challenges, and surgical findings that could affect how surgery is managed.

Amyand and De Garengeot hernias are good examples of this topic. In both types, the appendix is located inside the hernia sac, but the anatomical site differs: it is inguinal in the Amyand hernia and femoral in the De Garengeot hernia. The clinical and surgical importance also varies depending on the condition of the appendix. In some cases, the appendix is normal or only slightly inflamed, while in others it is gangrenous, perforated, or linked to abscess formation [2,11,12,35-39].

This variation matters because simply having the appendix present does not dictate management. Across the reviewed reports, the operative approach varied according to the extent of inflammation and contamination. In reported cases where the operative field was clean, mesh repair was used in selected patients. However, when gangrene, perforation, abscess, or contamination was present, appendectomy and tissue-based repair without permanent mesh were more frequently described. This shows that there is no single operative algorithm and that most of the available evidence comes from isolated reports instead of comparative studies [2,11,12,36-41]. The main operative decision points, particularly regarding mesh use and additional procedures, are presented in Table 3.

**Table 3:** Operative decision points for mesh use and additional procedures

Intraoperative finding	Main concern	Reported management tendency	Reported mesh-use pattern	Relevant entities	References
Normal appendix within hernia sac	Incidental appendix without infection	Hernia repair with or without appendectomy, depending on intraoperative judgement	Mesh was reported in selected cases without contamination	Amyand hernia, De Garengeot hernia	[1,43]
Inflamed appendix without perforation	Local inflammation and possible contamination	Appendectomy and hernia repair	Mesh use individualized; minimally invasive or combined approaches reported in selected cases	Amyand hernia, De Garengeot hernia	[7,14,19,21,23,24,35,40,41]
Gangrenous or perforated appendix	Contaminated or infected operative field	Appendectomy, drainage when required, and hernia repair	Permanent mesh was generally avoided or deferred in reports involving contaminated fields	Amyand hernia, De Garengeot hernia	[2,11,12,36–39]
Meckel’s diverticulum within hernia sac	Possible ischemia, necrosis, or atypical obstruction	Diverticulectomy or segmental small bowel resection according to bowel viability	Mesh use varied according to contamination and bowel condition.	Littré hernia	[5,26–30]
Colon or cecum within sliding hernia	Risk of bowel ischemia, perforation, abscess, or injury during dissection	Careful dissection; bowel resection in complicated cases	Mesh avoided or delayed when perforation or infection is present	Sliding inguinal hernia	[3,10]
Urinary bladder involvement	Risk of bladder injury; possible obstructive uropathy	Careful reduction and repair; minimally invasive or robotic approaches described in selected cases	Mesh was reported in selected cases with a clean operative field.	Sliding inguinal hernia	[3]
Persistent obstruction after apparent hernia reduction	Missed reduction en masse	Urgent open or laparoscopic exploration and bowel viability assessment	Mesh decision depends on bowel viability and contamination	Reduction en masse	[8,9,33,34,44]

Preoperative diagnosis is still challenging because these rare entities often resemble incarcerated or strangulated inguinal or femoral hernias. CT or ultrasonography can sometimes reveal unusual sac contents in certain cases, but diagnosis is still often made during surgery [4,6,7,21,30,33,34,42,43].

Littré hernias are distinct from hernias that contain the appendix because the main structure involved is Meckel's diverticulum, not the appendix. This leads to a different diagnostic challenge. Meckel's diverticulum, being a blind pouch that comes off the ileum, may become incarcerated without causing the usual signs of bowel obstruction. Because of this, the clinical presentation can look similar to a typical incarcerated groin hernia, and the actual contents of the hernia sac might only be discovered during surgery. Reported management differed according to the viability of the diverticulum and adjacent bowel. Diverticulectomy was described in some cases, whereas segmental small-bowel resection was reported when ischemia, necrosis, or bowel damage was present [5,26-30].

Sliding inguinal hernias create a different type of anatomical risk. In these cases, the colon or urinary bladder may form part of the hernia sac wall, rather than simply lying inside the sac. This distinction is important because unrecognized sliding anatomy may increase the risk of bladder or bowel injury during dissection. Bladder involvement may also have functional consequences. In one reported case, displacement of the urinary bladder was associated with hydronephrosis, hydroureter, and post-renal acute kidney injury [4].

Reduction en masse is a distinct situation, since the primary issue is not an unusual amount of visible sac content but rather the ongoing entrapment that persists after what seems to be a successful reduction. The swelling in the groin can go away, yet the bowel may still be stuck within the preperitoneal space, and the signs of obstruction can persist. This can lead to a delayed diagnosis if the recent history of either spontaneous or manual reduction is overlooked. CT scanning is especially valuable here, as it can reveal the incarcerated bowel loop inside the preperitoneal hernia sac [8,33,34,44].

Overall, the entities discussed in this study indicate that rare groin hernias should be considered not just as unusual case reports but as anatomical variations that can influence surgical decision-making. Hernias containing the appendix mainly bring up questions about whether to perform an appendectomy, the risk of contamination, and the use of mesh. Littré hernias prompt considerations about whether to do a diverticulectomy or a bowel resection. Sliding hernias require surgeons to recognize the possibility of organ involvement in the sac wall, while reduction en masse means there should be suspicion of ongoing obstruction even after the hernia reduction. These distinctions support the value of an anatomy-based approach to both diagnosis and operative planning [3-5,8-10,26-34,44].

**Figure 2:**
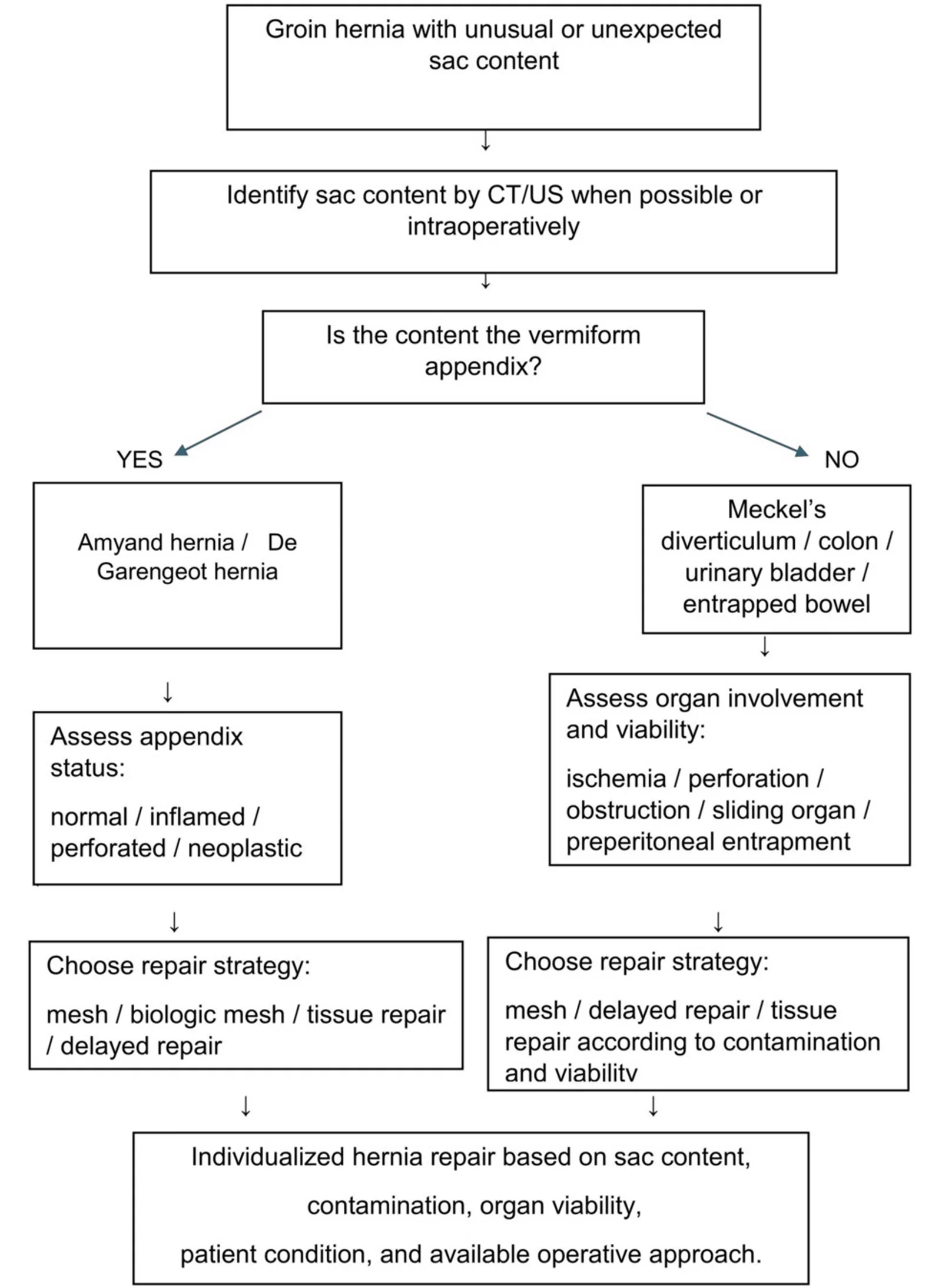
Proposed anatomy-driven decision pathway for rare groin hernias with unusual sac contents This pathway is not intended as a validated clinical algorithm or formal guideline, but as a proposed conceptual synthesis of the anatomical and surgical considerations derived predominantly from case reports and small case series included in this review. Image credits: Christos-Pavlos Filippou

Based on the qualitative synthesis of the reports included, an anatomy-driven decision pathway is shown in Figure 2. This pathway is not meant to serve as a formal guideline. Instead, it summarizes recurring anatomical and operative decision points reported in the reviewed cases, including sac content, inflammation or contamination, organ viability, and sliding or preperitoneal anatomy.

This review has several important limitations. Most of the included studies are case reports or small case series, which weakens the strength of the conclusions and prevents firm treatment recommendations. The reports also vary in how they describe imaging findings, surgical technique, mesh use, contamination, bowel viability, and postoperative outcomes. Because of this variation, it is difficult to directly compare cases, and a quantitative meta-analysis was not suitable. In addition, the literature search was restricted to PubMed/MEDLINE, English-language publications, and the predefined 2021-2026 publication period. Therefore, relevant case reports published in other databases, non-indexed journals, non-English-language sources, or outside the predefined time window may not have been captured. As a result, the methodological quality of the evidence was not evaluated with a validated, standardized instrument, which should be considered when interpreting the conclusions of this review. The absence of a formal risk-of-bias assessment is acknowledged as a limitation.

Future studies should aim to report rare groin hernias in a more standardized manner. Key details to include are the precise hernia type, sac content, preoperative imaging findings, degree of inflammation or contamination, surgical approach, whether mesh was used or avoided, and whether appendectomy, diverticulectomy, or bowel resection was needed, as well as postoperative outcome. More consistent reporting would make it easier to compare cases and could help develop practical decision-making pathways for surgeons. Accordingly, the available literature supports only descriptive, case-based observations, with reported management which vary according to sac content, contamination, organ viability, patient condition, and surgical expertise. The main limitations of the current evidence and suggested directions for future research are listed in Table 4.

**Table 4:** Evidence limitations and future research directions CT: computed tomography

Area	Findings from the reviewed literature	Main limitation	Future research direction	References
Study design	The available studies include case reports and small case series	Low level of evidence and limited generalizability	Multicenter case series or registry-based studies	[1,2,38]
Preoperative diagnosis	CT and ultrasonography may identify unusual sac contents in selected cases	Diagnosis is still frequently made during surgery	Standardized reporting of imaging findings for rare groin hernias	[7,20,26,38]
Surgical management	Open, laparoscopic, robotic, hybrid, mesh, non-mesh, and biologic mesh approaches are described	No uniform operative algorithm	Consensus-based decision pathways stratified by sac content, inflammation, contamination, and bowel viability	[3,22,23,36]
Mesh use	Mesh is used in selected clean cases; tissue or deferred mesh is reported in contaminated fields; biologic mesh is also described	Lack of comparative evidence on infection, recurrence, and long-term outcomes	Comparative studies of mesh, biologic mesh, and tissue repair in contaminated or potentially contaminated fields	[1,21,22,24,36]
Appendix-containing hernias	Appendix may be normal, inflamed, gangrenous, perforated, or rarely neoplastic	No clear consensus on management of a normal appendix or on mesh use with inflammation	Standardized reporting according to appendix status, contamination, and surgical approach	[1,2,14,21,43]
Littré hernia	Meckel’s diverticulum may be present without classical bowel obstruction	Very limited adult data and difficult preoperative diagnosis	Better characterization of imaging signs and criteria for diverticulectomy versus bowel resection	[26,27,29,30]
Sliding hernias	Colon or urinary bladder may form part of the sac wall	Risk of organ injury if anatomy is not anticipated	Studies focused on preoperative recognition and operative safety	[3]
Reduction en masse	Obstruction may persist despite apparent disappearance of the groin swelling	Diagnosis may be delayed if history of reduction is missed	Increased awareness and standardized evaluation after persistent symptoms following hernia reduction	[33,34,44]

## Conclusions

Rare inguinal and femoral hernias may contain unusual structures in the sac, yet they can lead to significant challenges in both diagnosis and surgery. In the cases reviewed, the hernia sac held organs such as the appendix, Meckel's diverticulum, colon, urinary bladder, or bowel that stayed trapped even after what seemed to be a successful reduction. These situations may require changes to the surgical plan, particularly when there is inflammation, perforation, contamination, bowel ischemia, or when the bladder is involved.

It is often not easy to diagnose these cases before surgery. Many patients arrive with symptoms that resemble those of typical incarcerated or strangulated groin hernias. While CT scans or ultrasound can sometimes help in selected situations, several published reports indicate that the true diagnosis is usually made during the operation itself. Because of this, surgeons need to be ready to encounter unexpected contents in the hernia sac when performing emergency or complex groin hernia repairs.

The choice of surgical technique should be based on what is found during the operation. Procedures such as appendectomy, diverticulectomy, bowel resection, careful dissection of the bladder or colon, tissue repair, mesh repair, or choosing not to use mesh may all be necessary depending on the findings. As most of the current evidence comes from case reports and small series, it is not possible to make firm treatment recommendations. More thorough reporting of future cases would help improve understanding of diagnosis, surgical decisions, and outcomes after surgery. The main value of this review is that it organizes these rare cases by sac content, diagnostic challenges, and anatomical findings that affect management, rather than just listing them as individual case reports.
